# Endolymphatic Hydrops in Patients With Intralabyrinthine Schwannomas

**DOI:** 10.3389/fsurg.2020.623078

**Published:** 2021-02-04

**Authors:** Yibo Zhang, Feitian Li, Chunfu Dai, Wuqing Wang

**Affiliations:** ^1^Department of Otology and Skull Base Surgery, Eye, Ear, Nose, and Throat Hospital, Fudan University, Shanghai, China; ^2^Key Laboratory of Hearing Medicine, Ministry of Health, Eye, Ear, Nose, and Throat Hospital, Fudan University, Shanghai, China

**Keywords:** intralabyrinthine schwannomas, endolymphatic hydrops, vertigo, hearing loss, MRI

## Abstract

**Purpose:** The presence of endolymphatic hydrops (EH) in patients with intralabyrinthine schwannomas (ILSs) is poorly understood. This study aims to determine whether there is a correlation between endolymphatic hydrops and clinical presentations of ILS.

**Methods:** Data from nine patients with ILSs were retrospectively reviewed between 2007 and 2020. Temporal bone MRI with intratympanic or intravenous injection of gadolinium was applied to detect ILSs and EH.

**Results:** 3D real inversion recovery (IR) sequence MRI of the temporal bone confirmed ipsilateral EH in four patients (4/6). All four patients with EH on MRI presented with vertigo similar to Meniere's disease. Among these patients with EH, one patient with EH in the cochlea showed moderate sensorineural hearing loss, while three patients with EH in both the vestibule and cochlea showed profound hearing loss. MRI demonstrated a transmacular tumor (TMA) in one patient, intravestibular (IV) in four patients, and vestibulocochlear (VC) in four patients. Two IV cases showed moderated hearing loss, while the TMA and VC cases showed profound hearing loss. Transotic resection of the tumor was applied in five patients; translabyrinthine resection was applied in one patient; two patients were under observation; and one patient was given intratympanic injection of gentamicin (ITG). During follow-up, all of the treated patients reported relief of vertigo, and postoperative MRI was performed in two patients, which showed no tumor recurrence. The two patients under observation showed no deterioration of hearing loss or vertigo. One patient was lost to follow-up.

**Conclusion:** EH concurrent with ILSs has been underestimated previously. With the extensive application of temporal bone MRI paradigms, such as 3D-real IR sequence MRI, more cases of potential EH in patients with ILS will be identified. The severity of hearing loss may be associated with the location of the tumor and the degree of EH.

## Introduction

Intralabyrinthine schwannomas (ILSs) are rare benign tumors within the membranous labyrinth that are reported to arise from the distal branches of the cochlear, superior vestibular, or inferior vestibular nerves (1, 2). Mayer described the first ILS case in 1917 (3). ILSs have been historically underdiagnosed, and most of them were unexpectedly identified during labyrinthectomy or cadaver autopsy (4, 5). With the application of magnetic resonance imaging (MRI), an increasing number of ILSs have been identified at the early stage.

Unilateral progressive hearing loss and recurrent vertigo are the most common symptoms in patients with ILSs (6, 7). These symptoms may mimic patients suffering from other inner ear disorders, such as Meniere's disease, and result in similar findings in acoustic and vestibular function tests, which further lead to the misdiagnosis of ILS at the early stage.

Endolymphatic hydrops (EH) is considered a pathological hallmark of Meniere's disease. However, EH can also be detected in other inner ear diseases, such as delayed endolymphatic hydrops, endolymphatic sac tumors, extralabyrinthine and intralabyrinthine schwannomas (8–10). Shinji Naganawa et al. demonstrated EH in some patients with vestibular schwannomas and concluded that there was no significant correlation between vertigo and vestibular hydrops. However, their cases mainly consisted of extralabyrinthine schwannomas. Anatomically, intralabyrinthine tumors are more likely to result in EH because of the possible mechanical blockade of the endolymph fluid (11). Only a few studies have reported EH in intralabyrinthine schwannomas (12–14), and the correlation between clinical symptoms and EH in ILS is still unclear. In addition, the underlying mechanism of ILS-associated EH remains unknown.

In this study, we retrospectively reviewed the medical records of nine patients with ILS and aimed to (1) demonstrate the occurrence of EH in ILSs; (2) reveal the correlation between clinical symptoms and imaging features of endolymphatic hydrops; and (3) discuss the diagnostic pitfalls of ILS.

## Methods and Patients

### Patients

The clinical data of nine patients with ILS who were treated at the Otology & Skull Base Surgery Department, Fudan University, between 2007 and 2020, were retrospectively reviewed. We retrieved data on age, symptoms, pure tone test, imaging, and management.

The diagnosis in six patients with ILS was confirmed by pathological examination postoperatively, while the other three patients who chose a wait-and-see strategy were diagnosed based on imaging. Six patients underwent both enhanced temporal bone MRI and temporal bone MRI with intratympanic or intravenous injection of gadolinium to detect EH. Patients 1, 2, and 3 only underwent enhanced temporal bone MRI, since MRI with intratympanic or intravenous injection of gadolinium was unavailable at that time in our hospital. According to the anatomic classification developed by Salzman et al. (7), ILSs in the present study are divided into six types, including intracochlear, transmodiolar, intravestibular, transmacular, vestibulocochlear, and transotic types. The tumor size was measured at its largest dimension on MRI.

### MRI Acquisition

For the intratympanic injection (IT) method, the patients received bilateral intratympanic injections of gadolinium diluted in saline (v/v 1:7) using a 22-gauge spinal needle and a 1-ml syringe. Gadopentetate dimeglumine was chosen in this research as the gadolinium contrast agent. Following the injections, the patients maintained an upright seated position for 30 min without speaking or swallowing. After 24 h, MRI scans were performed using a 3 T MR unit (Verio, Siemens Healthcare, Erlangen, Germany) with a 32-channel phased-array receive-only head coil. T2-space, 3D-real IR, and 3D-FLAIR sequence MRI images were collected. Briefly, the parameters for the 3D-real IR sequence were as follows: voxel size of 0.4 × 0.4 × 0.8 mm, scan time of 14 min, repetition time (TR) of 9,000 ms, echo time (TE) of 181 ms, inversion time (TI) of 1,730 ms, slice thickness of 0.80 mm, field of view (FOV) of 160 × 160 mm, and matrix size of 3,300 × 918. The parameters for the 3D-FLAIR sequence were as follows: voxel size of 0.7 × 0.7 × 0.6 mm, scan time of 6 min, TR of 6,000 ms, TE of 387 ms, TI of 2,100 ms, slice thickness of 0.60 mm, echo train length of 173, FOV of 220 × 220 mm, and matrix size of 1,701 × 810.

For the intravenous (IV) method, the patients received an intravenous injection of a double dose (0.4 ml/kg body weight) of Gd-HP-DO3A; 4 h later, MRI was performed. All scans were performed on a 3 T MRI scanner (Verio; Siemens Healthcare, Erlangen, Germany) using a 32-channel phased array receive-only coil. T2-space and 3D real-IR sequence MRI scans were applied to collect images. The parameters for the 3D real-IR sequence were as follows: voxel size of 0.17 × 0.17 × 0.6 mm, scan time of 15 min and 20 s, repetition time of 6,000 ms, echo time of 181 ms, inversion time of 1,850 ms, slice thickness of 0.6 mm, field of view of 160 × 160 mm, and matrix size of 768 × 768.

## Results

### Clinical Characteristics

The clinical characteristics of the study cohort are summarized in Table 1. The age of patients at presentation ranged from 35 to 69 years old (50.9 years old on average), and the sex ratio was 2:7 (male to female). Ipsilateral sensorineural hearing loss was observed in all patients. Seven patients presented with profound hearing loss, one patient showed moderate progressive hearing loss, and one patient demonstrated moderate sudden hearing loss. All patients experienced recurrent vertigo attacks with various characteristics (Table 2). All patients underwent caloric test, which showed ipsilateral weakness consistent with the tumor side.

**Table 1:** Clinical features of the study population.

**Patient**	**Age**	**Duration (years)**	**Tumor side**	**Symptoms**	**Caloric tests weaker side**	**Tentative diagnosis**	**Final diagnosis**
1	44–46	20	R	Profound pSNHL, V,T	R	Sensorineural hearing loss	ILS
2	35–37	4	L	Moderate pSNHL, V, T	L	MD	ILS
3	58–60	10	R	Profound pSNHL, V, T	R	Delayed endolymphatic hydrops	ILS
4	34–36	2	R	Moderate sudden SNHL, EF, V, T	R	Idiopathic hearing loss	ILS
5	68–70	1	R	Profound pSNHL, V, T	R	MD	ILS
6	36–38	12	R	Profound pSNHL, V, T	R	MD	ILS
7	58–60	30	L	Profound pSNHL, V, T	L	Depression, MD	ILS
8	61–63	10	R	Profound pSNHL, V, T	R	ILS	ILS
9	55–57	5	R	Profound pSNHL, V	R	ILS	ILS

*pSNHL, progressive sensorineural hearing loss; V, vertigo; T, tinnitus; EF, ear fullness*.

**Table 2:** Correlation between vertigo attacks and magnetic resonance imaging (MRI) presentation.

**Patient**	**Classification by Salzman et al**.	**Tumor size (mm)**	**Endolymphatic hydrops**	**Characteristics of vertigo**	**Duration per attack**	**Follow-up (months)**
1	TMA	5.0	Only enhanced MRI	Positional vertigo	Seconds to minutes	Lost
2	IV	3.0	Only enhanced MRI	Recurrent vertigo	Several hours	79
3	IV	1.5	Only enhanced MRI	Recurrent vertigo	10 min to several hours	106
4	IV	4.7	Cochlea (IV)	Recurrent vertigo	10 min to several hours	31
5	VC	3.4	Cochlea + vestibule (IV)	Recurrent vertigo	Several hours	7
6	IV	3.8	Cochlea + vestibule (IT)	Recurrent vertigo	Several hours	71
7	VC	2.5	Cochlea + vestibule (IT)	Recurrent vertigo	10 min	65
8	VC	4.6	No EH (IV)	Recurrent vertigo	Several minutes	8
9	VC	5.7	No EH (IV)	Drop attack	10 min to several hours	22

*TMA, transmacular; IV, intravestibular; VC, vestibulocochlear; EH, endolymphatic hydrops; IV, intravenous injection of gadolinium; IT, intratympanic injection of gadolinium*.

### Magnetic Resonance Imaging of Intralabyrinthine Schwannomas

**Figure 1:**
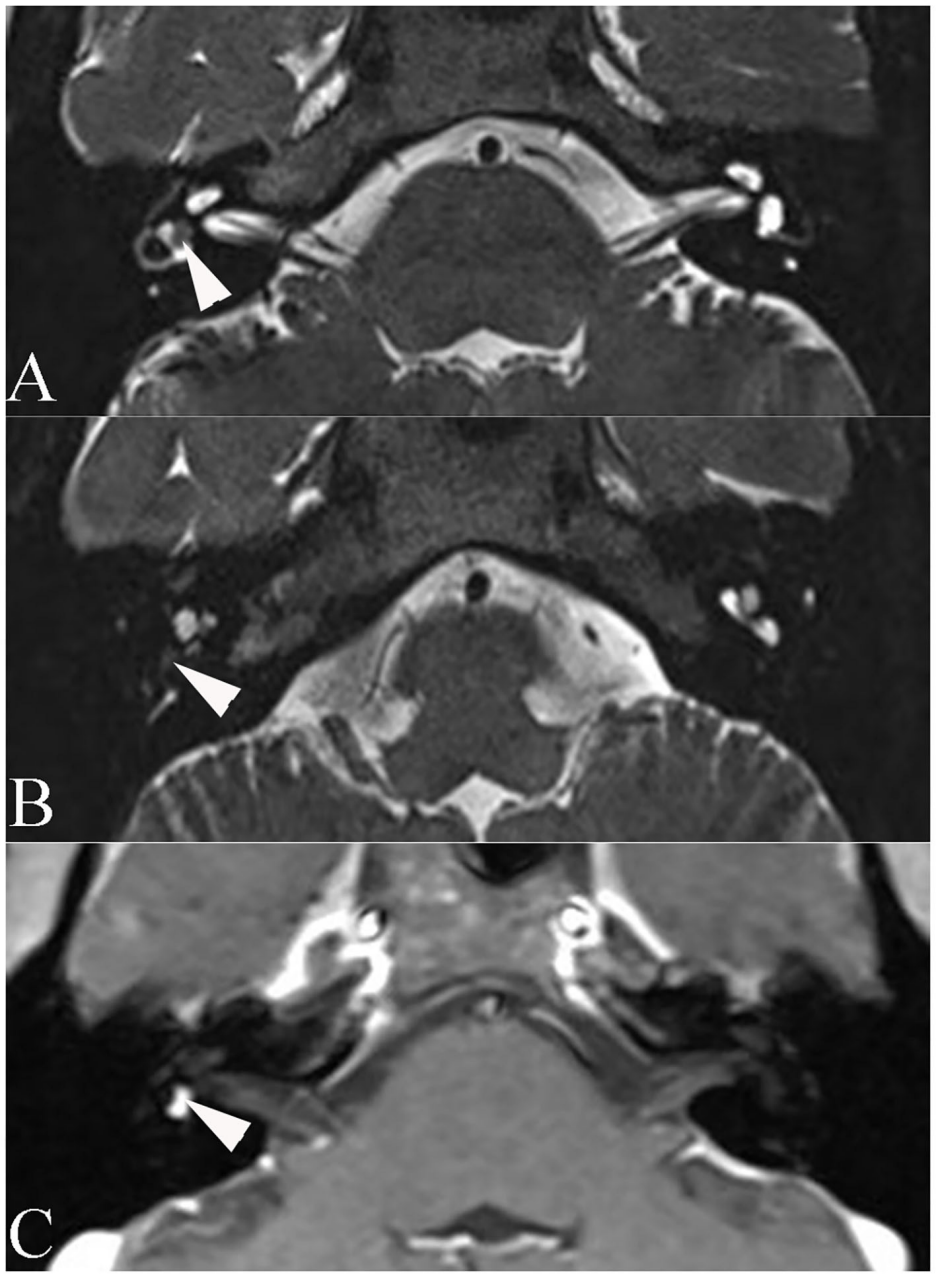
Axial magnetic resonance imaging of patient 6. **(A)** T2-weighted magnetic resonance imaging reveals a hypointense mass within the vestibule (arrowhead). **(B)** T2-weighted MRI reveals a hypointense mass within the basal turn of the cochlea (arrowhead). **(C)** Enhanced T1-weighted MRI reveals signal enhancement of the mass (arrowhead).

**Figure 2:**
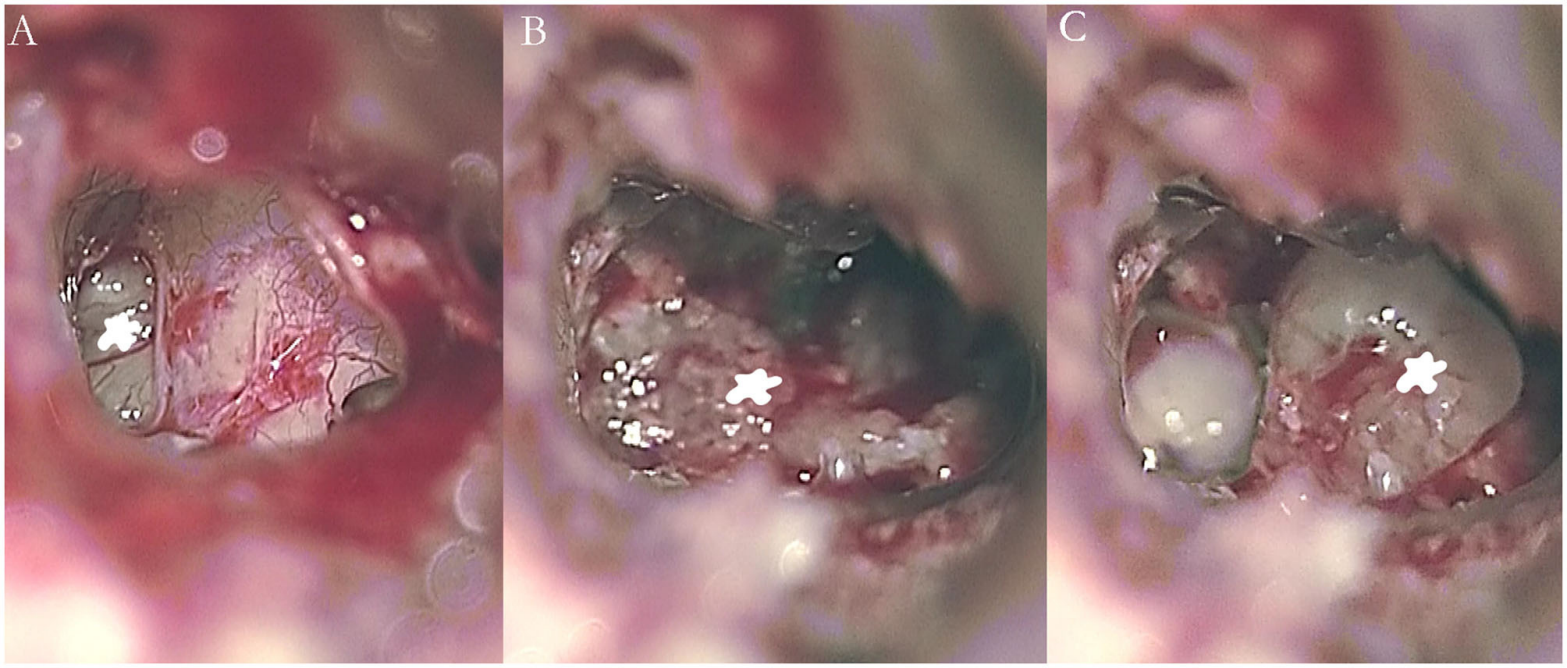
Intraoperative images showing an intralabyrinthine schwannoma in the right vestibule **(A, B)** and cochlea **(C)** of patient 5 (asterisk).

Temporal bone MRI with enhancement was performed in nine patients, which revealed a hypointense filling defect within the labyrinthine on T2-weighted images, and enhanced T1-weighted images revealed a homogeneously enhanced mass within the labyrinthine in these patients (Figure 1). Intraoperative findings confirmed the final diagnosis (Figure 2).

**Figure 3:**
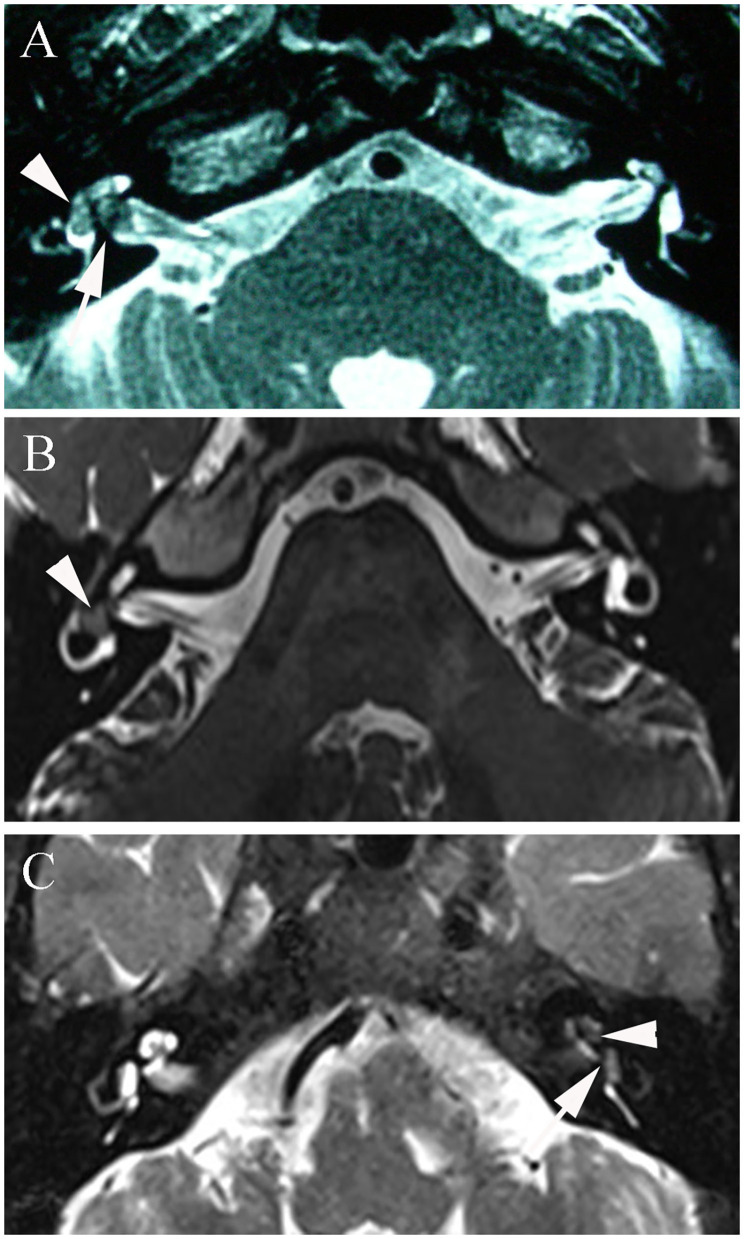
**(A)** T2-3D-space MRI of patient 1 with a transmacular tumor shows a hypointense mass in the vestibule (arrowhead) and a larger defect in the fundus of the internal auditory canal (arrow). **(B)** T2-3D-space MRI of patient 4 with an intravestibular tumor shows an isolated mass in the right vestibule (arrowhead). **(C)** Patient 7 with a vestibulocochlear tumor shows filling defects in both the vestibule (arrowhead) and cochlea (arrow) on T2-weighted MRI.

In the present study, MRI demonstrated a transmacular tumor in one patient, intravestibular tumors in four patients, and vestibulocochlear tumors in four patients (Figure 3). The mean tumor size in our study was 3.8 mm (Table 2).

### Endolymphatic Hydrops Concurrent With Intralabyrinthine Schwannomas

**Figure 4:**
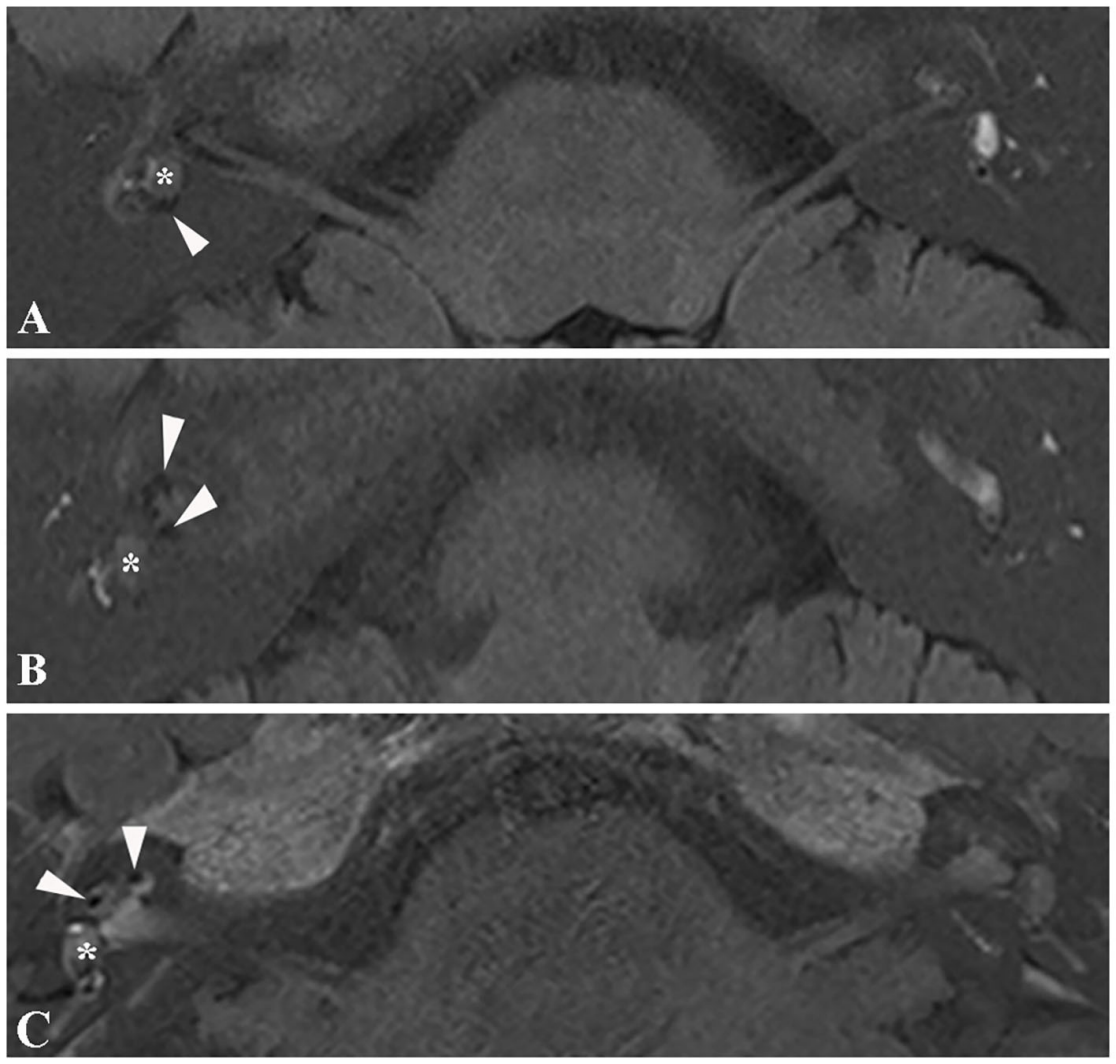
Magnetic resonance image of endolymphatic hydrops. **(A)** 3D real IR MRI of patient 6 shows endolymphatic hydrops in the vestibule (arrowhead) concurrent with an intravestibular tumor (asterisk). **(B)** 3D-real IR MR image of patient 6 with an intravestibular tumor (asterisk), presenting with endolymphatic hydrops in the cochlea (arrowhead). **(C)** 3D-real IR MRI of patient 4 with intravestibular tumor (asterisk) showing endolymphatic hydrops in the basal turn of the cochlea.

3D real inversion recovery (IR) sequence MRI of the temporal bone was applied in six patients to demonstrate endolymphatic hydrops, with the IV method applied in four patients and the IT method used in two patients. Four patients showed an intralabyrinthine hypointense mass concurrent with endolymphatic hydrops. Three patients presented with endolymphatic hydrops in both the cochlea and vestibule (Figures 4A,B), and one patient presented with endolymphatic hydrops in the cochlea (Figure 4C). All four patients with EH on MRI presented with vertigo similar to Meniere's disease.

### Surgical Outcomes

Five patients (patients 1, 3, 6, 7, and 8) with profound hearing loss underwent translabyrinthine resection of the tumor. Patient 2 with moderate hearing loss underwent surgery due to intractable vertigo. Patients 4 and 5 with serviceable hearing and tolerable vertigo chose conservative management. Intratympanic injection of low-dose gentamicin was used in patient 9 to relieve vertigo. No postoperative facial paresis, cerebrospinal fluid leakage, or other severe complications were encountered, except patient 7 who had delayed facial paresis 1 week after surgery and recovered completely in 1 month.

### Follow-Up

**Figure 5:**
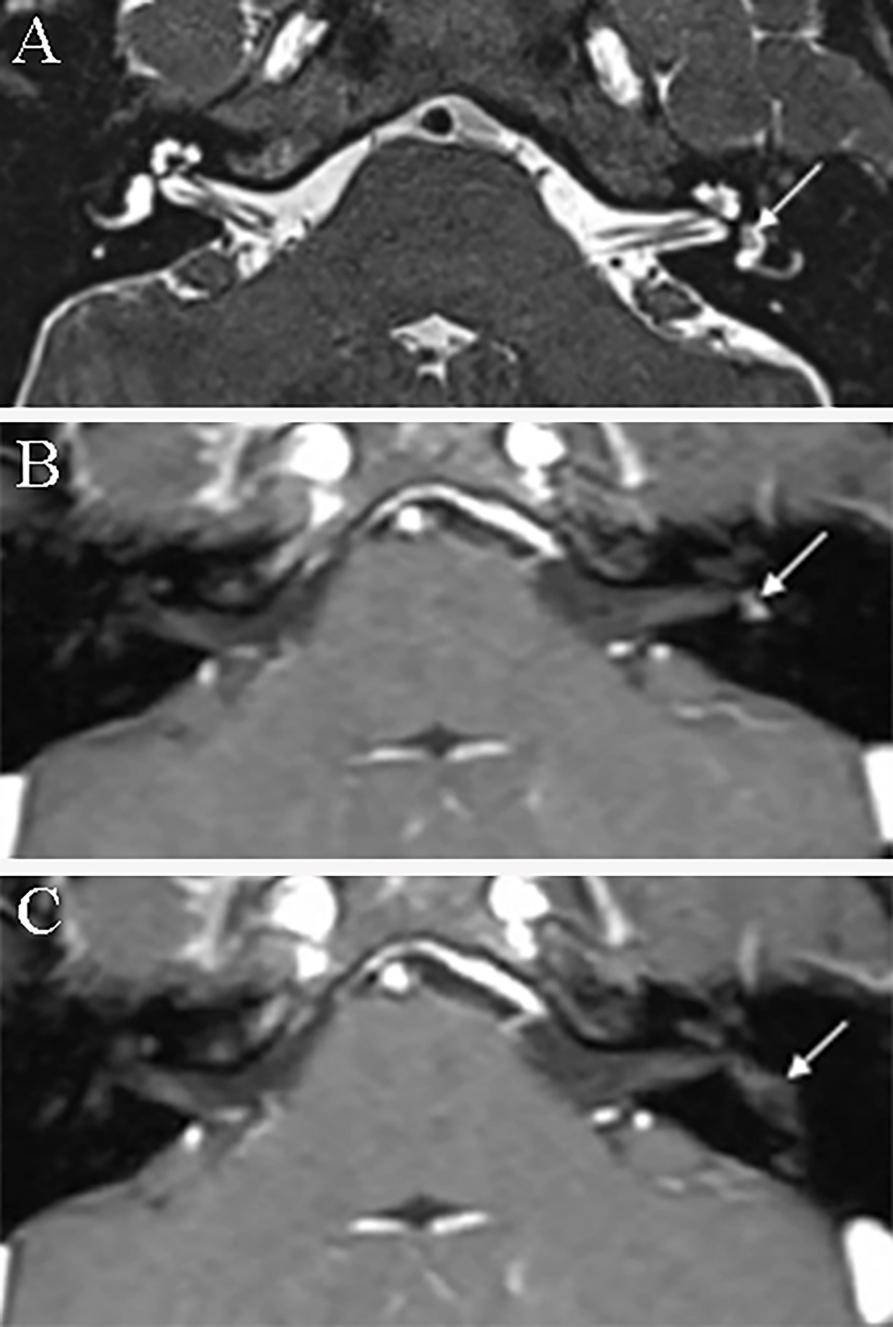
Axial magnetic resonance imaging of patient 4 before and after surgery. **(A)** T2-3D-space MRI reveals a hypointense filling defect within the left vestibule (arrow). **(B)** Enhanced T1-weighted MRI demonstrates a mass (arrow) within the vestibular cavity before surgery. **(C)** Enhanced T1-weighted MRI reveals no enhanced mass within the vestibule (arrowhead) of the tumor at 6 months postoperatively.

Eight patients were followed up. One patient was lost to follow-up. The mean follow-up time was 48.6 months (range, 7–106 months). During follow-up, five patients who underwent surgery reported no vertigo attacks, and two out of these five underwent MRI surveillance and did not show tumor recurrence (Figure 5). Two patients who were under observation and the patient who received ITG did not present with deterioration of hearing loss or vertigo.

## Discussion

### Prevalence

ILSs are rare disorders, but they may have been underestimated before. Van Abel et al. reported that the average delay between symptom onset and diagnosis of ILS was 7.0 ± 8.0 years (15). Currently, with the application of high-resolution MRI scans with or without contrast, more ILSs can be diagnosed at an early stage. It is estimated that the incidence of ILSs exceeds 1 per 100,000 person-years with modern diagnostic imaging (16). The rising incidence of ILSs in recent years most likely reflects an improved capacity for disease detection rather than a true increase in tumor development. According to the meta-analysis by Gosselin et al., no significant difference was found in the sex ratio of patients with ILS (17). Although the sex ratio was 2:7 (male to female) in the present study, no female predilection for this lesion could be drawn because of the limited sample size.

### Clinical Characteristics and Misdiagnosis of Intralabyrinthine Schwannomas

ILS is also known as primary inner ear schwannoma, since this term differentiates ILS from extralabyrinthine schwannomas that involve the labyrinthine, thus more precisely describing this lesion (15). Kennedy et al. proposed a new classification of ILSs in 2004 (18). On the basis of the Kennedy classification, Salzman et al. excluded the tympanolabyrinthine class because there were no observed cases fitting the description, and there was redundancy with the transotic subtype (7). Furthermore, Van Abel et al. proposed renaming intralabyrinthine schwannoma as primary inner ear schwannoma (PIES) to permit clear subsite categorization and modified the Kennedy classification, which excludes the transotic subtype and adds the translabyrinthine subtype (15). We applied the Salzman classification in this study because no translabyrinthine subtype cases were included. There is no consensus on the method of tumor size measurement, and previous studies have recorded the size of ILS based on MRI; however, a detailed description of the measurement was absent (2, 19). The sizes of ILSs measured in this study may be biased due to the natural growth pattern of ILSs. To determine the exact sizes of ILSs, a prospective study of 3D reconstruction and volumetric quantification of ILS needs to be performed in the future.

Sensorineural hearing loss is one of the most common symptoms of ILSs (20). It can occur as sudden or progressive non-recovering hearing loss. In the present study, moderate to profound sensorineural hearing loss was present in all cases, and the severity of hearing loss seemed to be related to the location of the tumor. Two intravestibular tumor cases showed moderated hearing loss, while all the transmacular and vestibulocochlear tumor cases showed profound hearing loss. Regarding the mechanism of hearing loss, Santos et al. performed a histopathologic study and demonstrated that, in addition to mechanical obstruction, the degeneration of hair cells, spiral ganglion neurons, and stria vascularis may also underlie the various SNHL in ILSs (21). Furthermore, hearing loss may also develop as a result of cochlear aperture obstruction and intralabyrinthine protein accumulation due to the tumor (22). All of our patients complained of tinnitus, which is consistent with a previous study (17).

Recurrent vertigo attacks, which mimic Meniere's disease, is another common symptom of ILSs, and combined with hearing loss and tinnitus, patients with ILS are prone to be misdiagnosed with MD at the early stage. Unfortunately, we still do not know if there are any typical differences in symptoms between patients with MD and patients with ILS, especially at the early stage. Though a differentiating clinical parameter between these two entities might be the extent of hearing loss. In ILS, frequently, total deafness occurs, whereas most MD cases end up with severe sensorineural hearing loss but not total deafness. A cohort study over a long period of time or a meta-analysis between the two groups could be meaningful to reveal the difference.

### Correlation Between Magnetic Resonance Imaging Characteristics and Vertigo in Patients With Intralabyrinthine Schwannomas

Jerin et al. reported two cases of ILS presenting with clinical symptoms that were similar to patients suffering from delayed endolymphatic hydrops, but no EH was demonstrated on temporal bone MRI in the two cases. A few studies have reported recurrent vertigo attacks in patients with ILS, mimicking Meniere's disease or delayed endolymphatic hydrops. However, few studies have shown that EH can be identified in patients with ILSs (12–14). Homann et al. first reported the coincidence of EH and intralabyrinthine tumors. However, due to the limited cases (two cases), it is unavailable to provide more information on the correlation of EH and the symptoms of ILS.

In the present study, the intralabyrinthine mass coexisting with EH was identified in four out of six patients using 3D real IR sequence MRI with injection of gadolinium. All four patients with EH on MRI presented with vertigo similar to Meniere's disease, so we assumed that EH is one typical imaging characteristic of ILSs, even in the early stage. It is hard to say whether EH or the intralabyrinthine mass is the first thing that can be detected on the MRI. Regarding the mechanism of EH in ILS, we speculated that the tumor may block the longitudinal flow of the endolymph, prevent the flow of the endolymph to the endolymphatic sac, or cause inflammation of the endolymph (23, 24).

In patient 1 with positional vertigo, temporal bone MRI with intratympanic injection of gadolinium revealed that the tumor was located in the vestibular cavity and the fundus of the internal acoustic meatus, and no EH was detected, which is consistent with the presentation. Slattery et al. reported a high proportion of patients displaying positional vertigo and assumed that it may be attributed to the direct effect of the tumor exerted on the cochlear and vestibular end organs (19).

In addition, the severity of hearing loss in the hydropic ears seems to be related to the site of hydrops, as hearing loss in patients with mild endolymphatic hydrops was moderate and profound in patients with both vestibular and cochlear hydrops. Further studies are needed to confirm the association between the symptoms and synchronous EH in ILSs.

Inner ear tumors are easily missed due to their occult location and small size, especially on conventional MRI. From our experience, it is not difficult to identify tumors and EH in the labyrinth of patients with ILS after careful review of high-resolution MRI using the IT or IV method. According to a previous study, there is no difference in the detection rate of EH by the IT or IV method (25). The strength of the IT method is the better contrast and resolution of the obtained pictures, but it is more invasive and could be affected by the external or middle ear condition. However, the IV method is less invasive and allows simultaneous monitoring of both ears (26). Thus, the IV method is now preferred in our hospital. High-resolution T2 MRI sequences are also recommended during follow-up but are sometimes refused by patients in whom vertigo does not recur.

### Management

Since vertigo is considered a risk factor for the growth of vestibular schwannomas (27), for patients with intractable vertigo or unserviceable hearing, resection of the tumor via the labyrinth or intratympanic injection of gentamicin is preferred (28). For patients with serviceable hearing or tolerable symptoms, a wait-and-see strategy is recommended. In recent years, endoscopic ear surgery has been widely used for its advantages, such as shortening the operation time, reducing the need for mastoidectomy, and reducing postoperative complications (29, 30). Studies have reported that endoscopic ear surgery can also be applied in treating ILS. Pan et al. reported the first documented case of endoscope-assisted resection of an intravestibular schwannoma and demonstrated that an endoscopic approach for tumor resection in the vestibule offered improved visualization of the vestibule compared to that in an operating microscope (31). Marchioni et al. reported a series of six cases affected by ILSs of the intracochlear type who underwent surgery with an endoscopic transcanal transpromontorial approach and demonstrated that the endoscopic approach should be preferred to other more invasive surgical techniques in patients with intracochlear ILSs (32). Ma et al. reported partial cochlectomy via a transcanal endoscopic approach, and simultaneous cochlear implantation was performed in an intracochlear schwannoma case, which resulted in good audiologic outcomes (33). Taken together, in addition to cholesteatoma removal and tympanoplasty, endoscopic ear surgery is also suitable for patients with ILS, owing to its direct access to hidden recesses, such as vestibules, small incisions, and lower risk of postoperative complications.

The limitation of this study is that the number of cases is small; thus, statistical analysis between symptoms and imaging presentations cannot be performed.

## Conclusion

EH concurrent with ILSs has been historically underestimated. With the extensive application of clinical MRI paradigms, such as 3D real IR sequence MRI, more potential cases of EH presenting in patients with ILS will be identified. The severity of hearing loss may be related to the location of the tumor and the degree of EH.

## Data Availability Statement

The original contributions presented in the study are included in the article/supplementary material, further inquiries can be directed to the corresponding author/s.

## Ethics Statement

The studies involving human participants were reviewed and approved by The medical ethics committee of the Eye, Ear, Nose, and Throat Hospital of Fudan University, Shanghai, China. The patients/participants provided their written informed consent to participate in this study.

## Author Contributions

YZ conceived the idea and drafted the manuscript, while FL collected the raw data and followed the patients. WW and CD generously provided the material and revised the manuscript. All authors contributed to the article and approved the submitted version.

## Conflict of Interest

The authors declare that the research was conducted in the absence of any commercial or financial relationships that could be construed as a potential conflict of interest.

## References

[1] Hamed A Linthicum FJ Intralabyrinthine schwannoma. Otol Neurotol. (2005) 26:1085–6. 10.1097/01.mao.0000185064.97210.01 16151363

[2] Neff BA Willcox TJ Sataloff RT Intralabyrinthine schwannomas. Otol Neurotol. (2003) 24:299–307. 10.1097/00129492-200303000-00028 12621348

[3] Mayer O Ein Fall von multiplen Tumoren in den Endansbreitungen des Akustikus. Z Ohrenheilk. (1917) 75: 95–113.

[4] Weymuller EJ Unsuspected intravestibular schwannoma. Arch Otolaryngol. (1975) 101: 630–2. 10.1001/archotol.1975.00780390044012 1080661

[5] DeLozier HL Gacek RR Dana ST Intralabyrinthine schwannoma. Ann Otol Rhinol Laryngol. (1979) 88:187–91. 10.1177/000348947908800207 443712

[6] Doyle KJ Brackmann DE Intralabyrinthine schwannomas. Otolaryngol Head Neck Surg. (1994) 110:517–23. 10.1177/019459989411000608 8208566

[7] Salzman KL Childs AM Davidson HC Kennedy RJ Shelton C Harnsberger HR. Intralabyrinthine schwannomas: imaging diagnosis and classification. Am J Neuroradiol. (2012) 33:104–9. 10.3174/ajnr.A2712 22158921 PMC7966166

[8] Butman JA Nduom E Kim HJ Lonser RR. Imaging detection of endolymphatic sac tumor-associated hydrops. J Neurosurg. (2013) 119:406–11. 10.3171/2013.2.JNS12608 23472846

[9] Naganawa S Kawai H Sone M Nakashima T Ikeda M. Endolympathic hydrops in patients with vestibular schwannoma: visualization by non-contrast-enhanced 3D FLAIR. Neuroradiology. (2011) 53:1009–15. 10.1007/s00234-010-0834-y 21221556

[10] Homann G Fahrendorf D Niederstadt T Nagelmann N Heindel W Lütkenhöner B. HR 3 Tesla MRI for the diagnosis of endolymphatic hydrops and differential diagnosis of inner ear tumors—demonstrated by two cases with similar symptoms. Rofo. (2014) 186:225–9. 10.1055/s-0033-1356221 24452492

[11] Schuknecht HF Rüther A. Blockage of longitudinal flow in endolymphatic hydrops. Eur Arch Otorhinolaryngol. (1991) 248(4):209–17. 10.1007/BF00173659 1859653

[12] Ekram T Koch SR Rajan J Intralabyrinthine schwannomas: review of anatomy, pathology, clinical features from an imaging perspective. J Clin and Diagn Res. (2012) 6:915–8.

[13] Jerin C Krause E Ertl-Wagner B Gürkov R. Clinical features of delayed endolymphatic hydrops and intralabyrinthine schwannoma: an imaging-confirmed comparative case series. English version. HNO. (2017) 65(Suppl 1):41–5. 10.1007/s00106-016-0199-6 27492473

[14] Venkatasamy A Bretz P Karol A Karch-Georges A Charpiot A Veillon F MRI of endolymphatic hydrops in patients with intralabyrinthine schwannomas: a case-controlled study using non-enhanced T2-weighted images at 3T. Eur Arch Otorhinolaryngol. (2020). 10.1007/s00405-020-06271-6. [Epub ahead of print]. 32770410

[15] Van Abel KM Carlson ML Link MJ Neff BA Beatty CW Lohse CM. Primary inner ear schwannomas: a case series and systematic review of the literature. Laryngoscope. (2013) 123:1957–66. 10.1002/lary.23928 23335152

[16] Marinelli JP Lohse CM Carlson ML. Incidence of intralabyrinthine schwannoma: a population-based study within the United States. Otol Neurotol. (2018) 39:1191–4. 10.1097/MAO.0000000000001875 29912836 PMC6131060

[17] Gosselin E Maniakas A Saliba I. Meta-analysis on the clinical outcomes in patients with intralabyrinthine schwannomas: conservative management vs. microsurgery. Eur Arch Otorhinolaryngol. (2016) 273:1357–67. 10.1007/s00405-015-3548-2 25673023

[18] Kennedy RJ Shelton C Salzman KL Davidson HC Harnsberger HR Intralabyrinthine schwannomas: diagnosis, management, and a new classification system. Otol Neurotol. (2004) 25:160–7. 10.1097/00129492-200403000-00014 15021777

[19] Slattery EL Babu SC Chole RA Zappia JJ. Intralabyrinthine schwannomas mimic cochleovestibular disease: symptoms from tumor mass effect in the labyrinth. Otol Neurotol. (2015) 36:167–71. 10.1097/MAO.0000000000000516 25111524

[20] Dubernard X Somers T Veros K Vincent C Franco-Vidal V Deguine O. Clinical presentation of intralabyrinthine schwannomas: a multicenter study of 110 cases. Otol Neurotol. (2014) 35:1641–9. 10.1097/MAO.0000000000000415 25098591

[21] Santos F Linthicum FH House JW Wilkinson EP. Histopathologic markers of hearing loss in intralabyrinthine schwannomas: implications for management. Otol Neurotol. (2011) 32:1542–7. 10.1097/MAO.0b013e318238fc63 22072265

[22] Asthagiri AR Vasquez RA Butman JA Wu T Morgan K Brewer CC. Mechanisms of hearing loss in neurofibromatosis type 2. PLoS ONE. (2012) 7:e46132. 10.1371/journal.pone.0046132 23049959 PMC3458837

[23] Takano S Iguchi H Sakamoto H Yamane H Anniko M. Blockage pattern of longitudinal flow in Meniere's disease. Acta Otolaryngol. (2013) 133:692–8. 10.3109/00016489.2013.771409 23768054 PMC3696340

[24] Yamane H Takayama M Sunami K Sakamoto H Imoto T Anniko M. Blockage of reuniting duct in Meniere's disease. Acta Otolaryngol. (2010) 130:233–9. 10.3109/00016480903096648 19585278

[25] Li Y Sha Y Wang F Lu P Liu X Sheng Y. Comprehensive comparison of MR image quality between intratympanic and intravenous gadolinium injection using 3D real IR sequences. Acta Otolaryngol. (2019) 139:659–64. 10.1080/00016489.2019.1600719 31130050

[26] Yamazaki M Naganawa S Tagaya M Kawai H Ikeda M Sone M. Comparison of contrast effect on the cochlear perilymph after intratympanic and intravenous gadolinium injection. AJNR Am J Neuroradiol. (2012) 33:773–8. 10.3174/ajnr.A2821 22173762 PMC8050462

[27] Artz JC Timmer FC Mulder JJ Cremers CW Graamans K. Predictors of future growth of sporadic vestibular schwannomas obtained by history and radiologic assessment of the tumor. Eur Arch Otorhinolaryngol. (2009) 266:641–6. 10.1007/s00405-008-0791-9 18704473

[28] Yang J Jia H Li G Huang M Zhu W Wang Z. Intratympanic gentamicin for small vestibular schwannomas with intractable vertigo. Otol Neurotol. (2018) 39:e699–703. 10.1097/MAO.0000000000001899 30020264

[29] Kiringoda R Kozin ED Lee DJ Outcomes in endoscopic ear surgery. Otolaryngol Clin North Am. (2016) 49:1271–90. 10.1016/j.otc.2016.05.008 27565392

[30] Marchioni D Rubini A Gazzini L Alicandri-Ciufelli M Molinari G Reale M Complications in endoscopic ear surgery. Otol Neurotol. (2018) 39:1012–7. 10.1097/MAO.0000000000001933 30113561

[31] Pan C Sewell A Michaelides E. Endoscope-assisted resection of intravestibular Schwannoma: a video case report. Laryngoscope. (2019) 129:986–8. 10.1002/lary.27605 30575039

[32] Marchioni D De Rossi S Soloperto D Presutti L Sacchetto L Rubini A. Intralabyrinthine schwannomas: a new surgical treatment. Eur Arch Otorhinolaryngol. (2018) 275:1095–102. 10.1007/s00405-018-4937-0 29560508

[33] Ma AK Patel N. Endoscope-assisted partial cochlectomy for intracochlear schwannoma with simultaneous cochlear implantation: a case report. Otol Neurotol. (2020) 41:334–8. 10.1097/MAO.0000000000002539 31923084

